# Pro-inflammatory Signaling in a 3D Organotypic Skin Model after Low LET Irradiation—NF-κB, COX-2 Activation, and Impact on Cell Differentiation

**DOI:** 10.3389/fimmu.2017.00082

**Published:** 2017-02-10

**Authors:** Anna Acheva, Giuseppe Schettino, Kevin M. Prise

**Affiliations:** ^1^Queen’s University Belfast, Centre for Cancer Research and Cell Biology, Belfast, UK; ^2^Section of Pathology, Department of Veterinary Biosciences, Faculty of Veterinary Medicine, University of Helsinki, Helsinki, Finland; ^3^National Physical Laboratory, Teddington, UK

**Keywords:** inflammation, COX-2, PGE2, ionizing radiation, 3D skin model

## Abstract

Nearly 85% of radiotherapy patients develop acute radiation dermatitis, which is an inflammatory reaction of the skin at the treatment field and in the surrounding area. The aims of this study were to unravel the mechanisms of radiation-induced inflammatory responses after localized irradiation in a human 3D organotypic skin culture model. This could provide possible inflammatory targets for reduction of skin side effects. 3D organotypic skin cultures were set up and locally irradiated with 225 kVp X-rays, using a combination of full exposure and partial shielding (50%) of the cultures. The secretion of pro-inflammatory cytokines, the phenotype, and the differentiation markers expression of the cultures were assessed up to 10 days postirradiation. The pro-inflammatory transcription factor nuclear factor kappa B (NF-κB) and cyclooxygenase-2 (COX-2) pathways have been studied. The results showed fast activation of NF-κB, most likely triggered by DNA damage in the irradiated cells, followed by upregulation of p38 MAPK and COX-2 in the irradiated and surrounding, non-irradiated, areas of the 3D cultures. The application of the COX-2 inhibitor sc-236 was effective at reducing the COX-2 mRNA levels 4 h postirradiation. The same inhibitor also suppressed the PGE2 secretion significantly 72 h after the treatment. The expression of a pro-inflammatory phenotype and abnormal differentiation markers of the cultures were also reduced. However, the use of an NF-κB inhibitor (Bay 11-7085) did not have the predicted positive effect on the cultures phenotype postirradiation. Radiation-induced pro-inflammatory responses have been observed in the 3D skin model. The activated signaling pathways involved NF-κB transcription factor and its downstream target COX-2. Further experiments aiming to suppress the inflammatory response *via* specific inhibitors showed that COX-2 is a suitable target for reduction of the normal skin inflammatory responses at radiotherapy, while NF-κB inhibition had detrimental effects on the 3D skin model development.

## Introduction

The skin is one of the most important and dose-limiting organs that is inevitably included in the exposed field during conventional radiotherapy. Due to the fast tissue turnover, the repair of radiation-induced DNA damage in the basal skin layer is often insufficient, which leads to high cell killing. Most affected are the hair follicle stem cells and melanocytes (1). After an initial decrease in cell number, there is an accelerated repopulation of the cells, which results in changes in the skin surface appearance (2). In addition, histamines are secreted and they induce a local pro-inflammatory response, the clinical result of which ranges from mild erythema to ulceration (2). After single doses, higher than 5 Gy, the skin reacts with an erythema-like response within a few hours including vasodilatation, edema, and leakage of plasma constituents from the capillaries. Erythema followed by dry and moist desquamation develops on second to third week after fractionated irradiation due to depletion of the stem cell compartment in the basal layer. Spreading out the dose over 6–8 weeks enables the skin to tolerate doses up to 60 Gy through stem cell repopulation (3, 4). After this period, the epidermis either heals or the changes progress to chronic wounds that might lead to necrosis (5). These effects develop at different levels during conventional radiotherapy. Depending on the individual sensitivity of the patient, they could cause complications, delay in radiation treatment, and even the need of surgical intervention. The late chronic reactions are reported to be permanent and progressive without complete treatment (6). This widely affects the quality of life of breast cancer patients (7, 8). Although numerous oral and topical treatments have been suggested, there is no generalized and satisfactory treatment of radiation-induced skin reactions (8). One of the main reasons is that mechanistic studies of skin reactions that involve all the stages at cellular, functional, and systemic level have been limited.

Interestingly, many of the routinely used or novel chemotherapy compounds (e.g., doxorubicin, docetaxel, paclitaxel, methotrexate, tamoxifen, etc.) have a synergistic effect on radiation-induced skin damage (6). The skin response also depends on extrinsic factors including radiation beam characteristics, dose fractionation schedule, affected volume, and surface area. In addition, many patients have concomitant disease that might enhance the effects of ionizing radiation (IR). Such conditions are diabetes mellitus, connective tissue diseases, radiosensitive genetic disorders such as ataxia telangiectasia, xeroderma pigmentosum, or Fanconi’s anemia, immunocompromised individuals, and obesity (6). Acute radiation-induced skin effects are always accompanied by a local inflammatory reaction. In the early stages of inflammation, there is triggering of pro-inflammatory cytokine production, which continues perpetually as a cascade during the whole process of cutaneous reaction development (6, 9). Cytokines play a very important role in local and distant signaling and orchestrate the interaction between different cell types at tissue and organ level. There is evidence that the major cytokines involved in the response of skin cells to IR are IL-1β, IL-6, TNF-α, and TGF-β; furthermore, the prostaglandin PGE2 and the chemokines IL-8 and eotaxin are involved (3, 4). Common for all the signaling molecules is their persistence in the cells and supernatants for 24–48 h postirradiation (9, 10). This persistence causes long-term inflammatory reactions in skin after irradiation that can lead to late effect fibrosis.

The COX enzyme is responsible for the conversion of arachidonic acid to prostanoids, which are secondary signaling molecules. It has two iso-forms: COX-1 that is constitutively expressed in the skin and cyclooxygenase 2 (COX-2) that is the inducible form produced after stimulation with cytokines and mitogens (11). COX-2 is known to be involved in the skin inflammation processes. It has also been found upregulated in conditions such as allergic asthma, rheumatoid arthritis, lipopolysaccharide- and TPA-induced skin inflammation, UVA- and UVB-induced erythema, etc. (11, 12). Numerous non-steroid anti-inflammatory drugs such as celecoxib, nimezulid, rofecoxib, sulindac, and sc-236, all with different selectivity of COX-2 over COX-1 inhibition, have been developed in order to suppress the inflammatory effects. Despite the highly promising initial results, some of these inhibitors showed side effects such as gastroulceritis, dyspepsia, renal failure, even cardiac infarction, and several of the early drugs, e.g., rofecoxib have been withdrawn for patient treatment after the initial clinical trials. However, some of the more recent ones, such as celecoxib and sc-236, are still regarded as promising anti-inflammatory drugs (13, 14).

Nuclear factor kappa B (NF-κB) is a transcription factor that can bind the kappa immunoglobulin-light chain enhancer (15). The NF-κB protein family consists of five members (p65, c-Rel, RelB, NF-κB1, and NF-κB2). Two of these, NF-κB1 and NF-κB2, are initially synthesized as larger proteins, and later, they are proteolytically cleaved to smaller DNA-binding functional units (p50 and p52, respectively). The NF-κB proteins form homo- and heterodimers activated from different intra- and extracellular stimuli as DNA double-strand breaks (DSB), TNF-α, IL-1β, LPS, etc. (16, 17). NF-κB proteins are kept inactive in the cytoplasm by the inhibitory subunit of the IκB (inhibitor of κB) family. IκB are a family of six proteins IκB-alpha, -beta, -epsilon, -gamma, -zeta, and Bcl-3, which mask the nuclear localization signal of the NF-κB. Upon phosphorylation, the IκB are degraded and the NF-κB is released for nuclear translocation (18).

Nuclear factor kappa B is reported to have an important role in inflammation and cancer. When activated, from various pro-inflammatory cytokines, NF-κB triggers the expression of genes responsible for cellular proliferation, antiapoptotic genes, and also has upregulatory function on angiogenesis. What is more, the transcription factor activation leads in turn to induction of cytokines responsible for immune reactions such as TNF-α, IL-1, IL-6, and IL-8, and also adhesion molecules which attract leukocytes to the sites of inflammation (15). Therefore, the dysregulation of this transcription factor is thought to be involved in various chronic inflammatory diseases, cancer development, and also in resistance to apoptosis-inducing cancer treatments (17). Interestingly, depending on the activating molecule, NF-κB could have either pro- or antiapoptotic effect (16). This specificity could be used in attempts to inhibit the NF-κB pathway in order to prevent cancer chemoresistance and to enhance the cancer cell killing at radiotherapy (19). The important role of NF-κB in the inflammation process makes this transcription factor a major target for treatment of chronic inflammatory diseases and inflammation-associated tumors (17). COX-2 and NF-κB have been ascribed roles in both cancer development and radioresistance of tumor cells (15, 20, 21). During radiotherapy, NF-κB is activated from DSB *via* the protein kinases ataxia telangiectasia mutated (ATM) and DNA-dependent protein kinase (DNA-PK) (15, 18). ATM is a serine kinase that senses DNA damage in the nucleus and, *via* different signal transduction pathways, regulates cell cycle, stress responses, and DNA repair (15). For example, patients with severe radiosensitivity, suffering from AT, have a defect in the ATM gene and additional NF-κB activation deficiency, which could be a reason for the enhanced apoptosis and severe responses to DNA-damaging agents (15). DNA-PK has a similar role in the DSB-mediated activation of NF-κB. In addition, the ROS generated during irradiation indirectly activate NF-κB *via* interactions with the allosteric regulators of the transcription factor (15). As a consequence, NF-κB has been attributed to a radioresistance-inducer role due to its antiapoptotic function (21). The use of various selective NF-κB inhibitors (such as dexamethasone) was suggested to be able to potentiate the neutralization of cancer cells after radiotherapy (15, 16, 21). The role of NF-κB inhibitors as enhancers of radiotherapy has also been widely described in the literature (21, 22). Inhibition of the NF-κB pro-inflammatory function would also be beneficial for patients as it could increase normal tissue sparing (21).

3D organotypic tissue cultures have been widely used to study cell differentiation, intercellular signaling, and the influence of tumor suppressors and sensitivity to cell death of certain tissue cell types [reviewed in Ref. (23)]. The models have been used for studying the effects of different chemical agents on the skin (24) and also for testing how different gene mutations or infectious diseases affect epidermal differentiation, morphology, and barrier function (25–27). Their spatial organization and functional properties make them suitable models for studying signaling processes *in vitro*. Thus, 3D skin cultures are a robust model for mechanistic studies on effects as radiodermatitis and testing of possible agents to reduce local inflammation and improve radiotherapy outcomes.

In this project, we aimed to investigate the pro-inflammatory reactions triggered in stratified 3D organotypic skin models post exposure to clinically relevant radiation doses. We used partial lead shielding in order to examine the signal spread from exposed to non-exposed areas. We focused on two main inflammation controlling molecules NF-κB and COX-2 and the cytokines involved in the signaling under their control. Finally, we tested the impact of inhibition of NF-κB and COX-2 function on radiation response and how this could mitigate the spread of pro-inflammatory signaling to the surrounding tissue.

## Materials and Methods

### Cell Culture

J2-3T3 mouse fibroblast cells were a kind gift from Prof. Dennis McCance laboratory (Queen’s University Belfast, Belfast, UK). They were cultured in 75 cm^2^ flasks at density 6.7 × 10^3^ cells/cm^2^ in DMEM (MP Biomedicals, Illkirch, France) supplemented with 10% FCS (PAA, Pasching, Austria) and 1% penicillin/streptomycin (PAA, Pasching, Austria), refed every third day and replated after reaching 80% confluency as assessed by microscope analysis.

N/TERT-1 normal human keratinocytes immortalized by transfection to express TERT (28, 29) obtained from Dr. Rheinwald from Harvard Institutes of Medicine, Boston, MA, USA, were grown in a medium commercially available from GIBCO, keratinocyte serum-free medium (K-sfm) (Invitrogen, Carlsbad, CA, USA). The medium has been supplemented as described in Ref. (29).

### Organotypic Raft Cultures

Organotypic raft cultures were set up according to the method described in Ref. (23, 25) with modifications as described below. For clarity, we will further refer to the organotypic raft cultures also as “3D skin model,” “3D raft cultures,” and “3D organotypic model.” J2-3T3 fibroblast cells were treated with mitomycin C (Sigma-Aldrich, St. Louis, MO, USA) (4 µg/ml) to block the mitosis for a minimum of 2 h before using them for raft cultures. Fibroblasts were then trypsinised, spun down, and added to 60–70% confluent N/TERT-1 keratinocytes in T25 flasks (~1:3 fibroblast:keratinocytes ratio). J2-3T3 were added to keratinocytes in E-medium [formulation described in Ref. (30)] + EGF (Calbiochem, La Jolla, CA, USA) (10 ng/ml) and cocultured overnight. Before the keratinocytes were used in the 3D cultures, the fibroblasts were trypsinised from the cocultures by 2–3 min incubation with trypsin solution, followed by washing with PBS. In a separate step, collagen Type I plugs containing J2-3T3 feeder cells were prepared from 3 mg/ml final concentration Rat tail collagen (acidic) (BD, Bedford, MA, USA), 10× DMEM (MP Biomedicals, Illkirch, France), few drops of filter sterilized 1M NaOH to neutralize the acidic collagen. The final volume was 2 ml per plug with a diameter of 23 mm. The collagen gels with added 4.5 × 10^5^ J2-3T3 cells were let to solidify in hanging membrane inserts (BD Falcon, NJ, USA) in 6-well plates. Once the gels have set, the 1 × 10^6^N/TERT-1 keratinocytes per plug were plated on top and allowed to attach for 1 h. The cultures were fed with E-medium + EGF. On the next day, medium from the top chamber was aspirated, and the cultures were fed from the bottom chamber with E-medium without EGF with the collagen gels at the air liquid interface to stimulate differentiation. The 3D skin cultures were fed daily for the first 2–3 days then every 2 days. The cultures were harvested at day 11, fixed in 4% paraformaldehyde for 1 h at room temperature, and processed for paraffin embedding, sectioning at 6 µm thick sections for immunofluorescence and H&E staining.

### Irradiation Experiments

Radiation exposures were performed using the XRAD 225 (225 kVp X-ray) from Precision X-rays Inc. (N. Branford, CT, USA) at a dose rate of 0.591 Gy/min measured with a calibrated 0.6 cm^3^ waterproof Farmer Ionization Chamber with an UNIDOS E measuring device (PTW, Grantham, Lincolnshire, UK). The irradiation experiments with half shielding of the 3D cultures were performed using custom-designed frame and 2 cm thick low melting point lead-containing alloy MCP-96 blocks (Par Scientific, Odense, Denmark) positioned 2.05 cm above the samples. The efficiency of shielding was confirmed by EBT3 Gafchromic^®^ film (Vertec Scientific Ltd., Reading, UK) measurement with less than 2.3% of the dose delivered reaching under the shielded area and a sharp transition (dose falls from 90 to 10% within 2 mm).

### COX-2 and NF-κB Inhibitors Treatment of the 3D Cultures

The COX-2 selective inhibitor sc-236 (Cayman Chemicals, Ann Arbor, MI, USA) was used to block the enzyme activity. The compound has IC_50_ of 10 nmol/l and approximately 18,000-fold COX-2 selectivity over COX-1, the other isoform of cyclooxygenase. It was dissolved in DMSO at 20 mmol/l stock, and this stock solution was stored frozen at −20°C.

Bay 11-7085 (Sigma-Aldrich, Gillingham, Dorset, UK) is a specific irreversible inhibitor of TNF-α-mediated IκB phosphorylation with an IC_50_ ~ 10 μmol/l. It was dissolved in DMSO at 20 mmol/l stock, and this stock solution was stored frozen at −20°C.

The N/TERT-1 keratinocytes and the 3D skin cultures were treated 1 h before irradiation. The inhibitors were kept in the culture medium for the whole duration of the experiment (up to 7 days for the differentiation assay).

### MTT Assay

The method is based on the reduction of the yellow tetrazole 3-(4,5-dimethylthiazol-2-yl)-2,5-diphenyltetrazolium bromide (MTT) by the mitochondrial dehydrogenases to purple formazan dye. Briefly, 5,000 cells per well were plated in 200 µl of medium on a 96-well plate. The cells were allowed to attach overnight and treated with COX-2 or NF-κB inhibitor for 72 h. For each inhibitor concentration, there were six replicate wells. A total of 20 µl of the 5 mg/ml tetrazolium MTT (Sigma-Aldrich, St. Louis, MO, USA) was added to each well and left for 3 h in an incubator at 37°C, 5% CO_2_ allowing the cells to metabolize the dye. The medium was removed and the formed crystals of purple formazan were dissolved with 150 µl isopropanol (Sigma-Aldrich, St. Louis, MO, USA). The plates were wrapped in aluminum foil and agitated on a shaker for 20 min. Absorption of the purple MTT solution was measured at 570 nm with a BioTrak II plate reader (Amersham Biosciences, supplied from Vector Scientific, UK). Cell viability was calculated after subtracting absorption of isopropanol, which was used as a blank by normalizing to the untreated control.

### qRT-PCR

Total RNA was isolated from the 3D raft cultures using TRIzol™ reagent (Invitrogen, Carlsbad, CA, USA). The frozen 3D skin specimens were cut into small pieces (approximately 50–100 mg) while still frozen. The pieces were immediately placed into the TRIzol reagent for homogenization and vortexed at maximal speed for 60 s. The samples were incubated at RT for 5–10 min after homogenization.

Chloroform extraction and RNA precipitation were performed as described in the TRIzol™ reagent (Invitrogen, Carlsbad, CA, USA) manufacturer’s instructions. The RNA concentration was measured on a Nanodrop spectrophotometer ND-1000 (Mason Technology, Dublin, Ireland). Purity of the RNA was monitored by calculating the A260/280 ratio for protein contamination and A260/230—for polysaccharide contamination. The A260/280 in all experiments was >2. The integrity of the isolated from the 3D organotypic cultures RNA has been checked up by running on denaturing agarose gels in earlier experiments.

DNase I treatment and the reverse transcription with M-MLV (Moloney murine leukemia virus) reverse transcriptase were accomplished using reagents from Invitrogen (Carlsbad, CA, USA) according to the manufacturer’s protocol.

The polymerase chain reaction was performed using COX-2 primers obtained from Qiagen (Mainz, Germany), assay number Hs_PTGS2_1_SG resulting in an amplicon length of 68 bp. The PCR efficiency was determined by calibration curves generated from serial dilutions of cDNA synthesized from non-treated 3D cultures (Figure S1 in Supplementary Material).

Each RT-PCR reaction contained 12.5 µl SYBR Green (Applied Biosystems, Carlsbad, CA, USA), 1 µl primer mix, 2.5 µl template, and 6.5 µl water. The RT-PCR reactions were run on DNA Engine Opticon 2 Real-Time Cycler (BioRad, Waltham, MA, USA). The following qRT-PCR protocol was used (1) one incubation step 50°C for 2 min; (2) one incubation step 95°C for 5 min; and (3) 40 cycles 95°C for 10 s; 60°C for 30 s; incubate 72°C for 5 min; melting curve 69–95°C; read every 0.2°C hold 1 s; 72°C for 5 min. For every condition, three independent experiments in triplicates of each sample were performed. A standard curve was generated from serial dilutions of cDNA synthesized from non-treated 3D cultures and used to manually set the threshold line to determine the threshold cycle (*C*_T_) values for COX-2 expression in the unknown samples. These results were normalized to the expression of the reference gene 18S rRNA in the same samples (Qiagen assay number Hs_RRN18S 1 SG; amplicon length 149 bp). 18S rRNA has been previously used as a reference gene in larger scale organotypic culture gene expression studies (31). The normalization was performed using the $2^{-\Delta \Delta C_{\text{T}}}$ method according the BioRad Opticon 2 Real-Time Cycler user manual. First, we normalized the *C*_T_ of the target gene to that of the reference (ref) gene, for both the test sample and the calibrator sample:

$$\Delta C_{\text{T}(\text{test})}=C_{\text{T}(\text{target},\text{ test})}-C_{\text{T}(\text{ref},\text{ test})}$$

$$\Delta C_{\text{T}(\text{calibrator})}=C_{\text{T}(\text{target},\text{ calibrator})}-C_{\text{T}(\text{ref},\text{ calibrator})}$$

After that, we normalized the Δ*C*_T_ of the test sample to the Δ*C*_T_ of the calibrator:

$$\Delta \Delta C_{\text{T}}=\Delta C_{\text{T}(\text{test})}-\Delta C_{\text{T}(\text{calibrator})}$$

Finally, we calculated the expression ratio:

$${\text{2}}^{-\Delta \Delta C_{\text{T}}}=\text{Normalized expression ratio}.$$

In the formulas, *C*_T_ are the threshold cycles and Δ*C*_T_ is the difference in the *C*_T_ values.

### Immunofluorescence Staining of 3D Organotypic Skin Cultures

Culture sections were deparaffinized with xylene and decreasing alcohol concentrations. Following that, sections were subjected to antigen unmasking where required [i.e., filaggrin (FLG)] with citrate buffer (20 min boiling, followed by 20 min on the bench). The slides were blocked in 10% FCS, 0.2% PBS-Triton X-100 for 30 min, and incubated with primary antibodies in the following dilutions: 1:50 for cytokeratin 1 (K1) (Vector Laboratories, Burlingame, CA, USA) and 1:100 for FLG (AnaSpec, San Jose, CA, USA). After overnight incubation with the primary antibody at room temperature, the sections were washed with 0.1% Triton X-100 in PBS and incubated with 1:1,500 secondary Alexa 488 conjugated antibody (Molecular Probes, Invitrogen, OR, USA) for 1 h at room temperature. After washing with washing buffer, slides were counterstained with DAPI-containing mounting medium Vectashield (Vector Laboratories, Burlingame, CA, USA) and sealed with clean nail varnish.

### Western Blotting

N/TERT-1 cultured in 2D or differentiated in 3D organotypic cultures (total cultures) were lysed with lysis buffer containing 50 mM Tris, HCl pH 8.0, 150 mM NaCl, 1% Triton X-100, and protease and phosphatase inhibitor cocktail (Roche, Mannheim, Germany). The protein concentration was measured according to the Bradford method (BioRad, Munich, Germany). The protein solution was diluted in NuPage loading buffer (Invitrogen, Carlsbad, CA, USA) and 30 or 60 µg per line loaded on 4–12% Bis-Tris NuPage precasted gels (Invitrogen, Carlsbad, CA, USA). The separated proteins were transferred on nitrocellulose membrane using iBlot semidry transfer apparatus (Invitrogen, Carlsbad, CA, USA). Membranes were blocked with blocking buffer [5% skimmed milk, 0.1% Tween 20 in PBS (PBS-T)] for 1 h at room temperature and incubated with primary antibody against COX-2 (Millipore, Temecula, CA, USA) 1:500; phospho-p65^Ser276^; phospho-p38^Tyr180/Tyr182^ (Cell Signaling, Denver, MA, USA) 1:1,000; GAPDH and β-actin 1:5,000 (Sigma-Aldrich, St. Louise, MO, USA) overnight at 4°C. The membranes were washed with 0.1% PBS-T and incubated with secondary HRP-conjugated antibody (ECL™ anti-mouse/anti-rabbit IgG, GE Healthcare, Little Chalfont, UK) for 1 h at room temperature. After washing with 0.1% PBS-T, membranes were incubated with SuperSignal ECL (Thermo Scientific, Rockford, IL, USA) and developed on X-ray sensitive film (GE Healthcare, Little Chalfont, UK).

### Quantification of Differentiation Marker Expression in 3D Cultures

3D culture differentiation analysis was performed by quantifying the total expression of the differentiation marker proteins and by measuring the thickness variation of the cornified layers. Pictures were taken under the Carl Zeiss Axiovert 200M (Carl Zeiss, Göttingen, Germany) inverted fluorescence microscope with a 63× oil objective and CCD camera using Axiovision Rel. 4.6 software, all from Carl Zeiss, Göttingen, Germany. The exposure parameters were kept constant, and the images were processed using Image J 1.04 (National Institutes of Health, Bethesda, MD, USA, http://imagej.nih.gov/ij/, 1997-2016) software. The percentage of K1 or FLG-positive area was quantified using thresholds set on the control images. The manual region of interest tool of Image J was used to determine the borders of the corresponding epidermal layer where the marker is expressed—granular layer (for FLG) or granular and suprabasal layer (for K1). Then, the intensity calculation option of the program was utilized to obtain the intensity per area values. The ratio between the treated and control samples intensity was used to represent graphically the expression. Results are mean from two independent experiments with two replicate slides per point and five visual fields with 145 µm length per each slide.

### Enzyme-Linked Immunosorbent Assay (ELISA)

The medium samples were collected at time points 0, 24, 48, and 72 h after irradiation of the 3D cultures. In part of the experiments, the 3D skin was pretreated with sc-236 and Bay 11-7085 at the concentrations described above. The inhibitors were added 1 h before irradiation and were present in the culture medium for the whole duration of the experiment. The collected medium samples were centrifuged for 5 min at 3000 *g* to pellet any debris present that could affect the analysis and stored at −20°C.

For measuring the concentration of the PGE2 in the 3D cultures medium, an ELISA kit Parameter™ (R&D Systems, Abingdon, UK) was used according to the manufacturer’s instructions.

### Statistical Analyses

Differences between groups were analyzed using a Student’s *t*-test or one-way ANOVA, Tukey posttest, part of the statistical package of GraphPad Prism version 6.00 for Windows, GraphPad Software, La Jolla, CA, USA, www.graphpad.com.

## Results

We analyzed the relative COX-2 expression in directly irradiated and shielded 3D skin cultures *via* qRT-PCR. The sham irradiated controls were collected together with the 24 h samples at the end of the experiment. The time points studied were chosen based on data from previous studies (32, 33).

**Figure 1:**
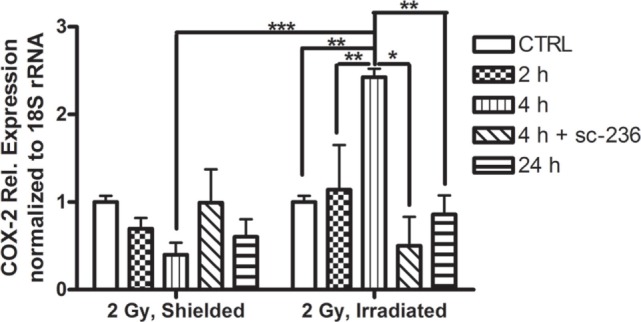
**Comparison of cyclooxygenase-2 mRNA expression in 3D organotypic cultures: control (CTRL), 2, 4, and 24 h after 2 Gy irradiation, and 4 h after 2 Gy irradiation plus 5 µmol/l sc-236 (*n* = 3)**. The CTRL samples, all non-irradiated, have been collected and analyzed at 24 h after the irradiation of the test samples. Statistical analysis, one-way ANOVA Tukey posttest; **p < 0.01; ***p < 0.001; *p < 0.05.

The results from the qRT-PCR confirmed previously reported upregulation of the COX-2 gene that starts 2 h postirradiation and further increases at 4 h postirradiation (Figure 1). Interestingly, the shielded areas had an opposite downregulation of mRNA levels for the 2- and 4-h time points. This downregulation could not be explained by a simple delay in the gene response in the non-directly exposed bystander cells, because no increase had been observed at the later time points (24 h).

### Changes in Radiation Response after Inhibition of COX-2 Signaling

After we observed significant COX-2 upregulation in the 3D skin model following localized irradiation, we tried to find ways to modify the radiation-induced responses of the model by altering the COX-2 expression and activity. For this purpose, we used sc-236, a well-described highly selective COX-2 inhibitor (20).

**Figure 2:**
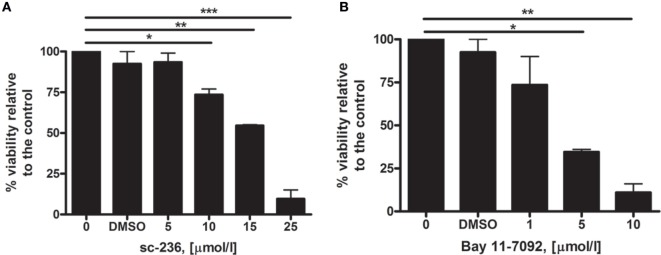
**Cytotoxicity testing of sc-236 cyclooxygenase-2 selective inhibitor (A) and Bay 11-7085 selective NF-κB inhibitor (B) *via* MTT assay 72 h after the treatment (*n* = 2)**. Error bars—SEM; statistical analysis—One-way ANOVA, Tukey posttest; *p < 0.05; **p < 0.01; ***p < 0.001.

Before applying the inhibitor on the 3D cultures, its toxicity on N/TERT-1 cells was tested using the MTT assay as described in Section “Materials and Methods.” The results for 5, 10, 15, and 25 µmol/l concentrations of the inhibitor incubated with 5,000 keratinocyte cells for 3 days indicated a statistically significant increase in the toxicity for concentrations ≥10 µmol/l (Figure 2A).

Accordingly from the results from the MTT assay and the data from the literature, we adopted 5 µmol/l as a working concentration for studying the long-term radiation-induced effects in the 3D cultures. The cultures were pretreated for 1 h with the sc-236 and its effect on the COX-2 expression was measured at 4 h postirradiation when the peak in COX-2 upregulation was observed (Figure 1).

At the 4 h time point, when in the directly irradiated samples, there was >2.5 times increase in the COX-2 mRNA levels, sc-236 pre-treatment of samples led to a statistically significant downregulation of the COX-2 gene expression to less than 0.5 of the control levels (Figure 1). The COX-2 mRNA levels in the shielded areas did not change significantly after application of the sc-236 inhibitor and were even slightly higher, than in the controls. This is probably due to the initial lack of activation of mRNA synthesis in the shielded areas (Figure 1) and the application of the inhibitor not suppressing the basal COX-2 mRNA levels.

### Morphology of the 3D Cultures after sc-236 Treatment

**Figure 3:**
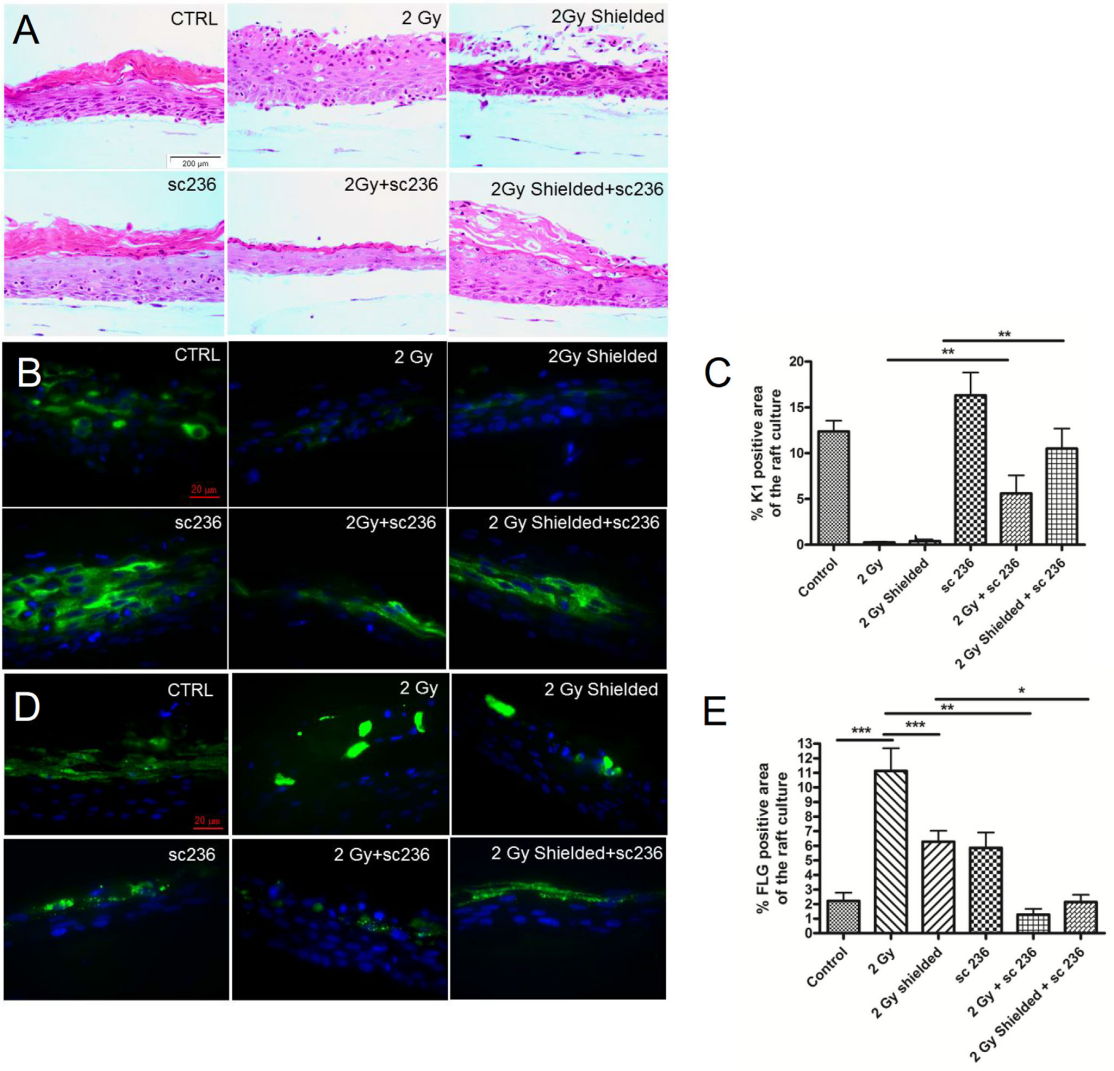
**Rescue of the normal morphology of the 3D cultures after irradiation in the presence of 5 µmol/l specific cyclooxygenase-2 inhibitor sc-236 7 days postirradiation**. **(A)** H&E staining; immunofluorescence staining of **(B)** cytokeratin 1 and filaggrin **(D)** as differentiation markers. Quantification of the differentiation maker expression by Image J as described in Section “Materials and Methods.” **(C)** and **(E)** Blue—DAPI; green—K1. Error bars—SEM; **p < 0.01; ***p < 0.001, one-way ANOVA analysis, Tukey posttest.

Irradiation with the clinically relevant dose of 2 Gy changed the morphological structure of the 3D skin model increasing the thickness of the cornified layer (Figure 3). The expression of both early (Cytokeratin 1) and late (FLG) differentiation markers were modified as K1 was downregulated and FLG upregulated (Figures 3B–E). Following the experiments with the sc-236 inhibitor and its effect on the pro-inflammatory COX-2 enzyme levels, a 5 µmol/l concentration was chosen to treat 3D skin cultures up to 7 days postirradiation to evaluate the effect on radiation-induced morphological changes (Figure 3).

Morphological analysis of the tissue sections treated with only sc-236 during the 7 days incubation period showed a normal tissue morphology, stratification, and thickness of the cornified layer. We compared the phenotype of half-shielded 3D cultures after the clinically relevant dose of 2 Gy radiation and half shielding with the same dose, but with addition of 5 µmol/l sc-236 treatment during the incubation period. The results were showing reduction of the hyperproliferation and formation of normal keratinized layer (Figure 3A).

In addition to the morphological analysis, we investigated the effects on stratification of the 3D model after the COX-2 inhibitor treatment through following differentiation in the 3D cultures and immunofluorescence of paraffin-embedded sections for expression of the early differentiation marker K1 (Figures 3B,C). There was a slight upregulation in the K1 expression from 5 µmol/l sc-236 treatment, but the quantification of the expression showed that the effect was not statistically significant (Figures 3B,C). There was also rescue of the K1 expression in the 2 Gy irradiated and sc-236 treated 3D cultures (Figures 3B,C) observed as statistically significant increases of the K1 levels in the shielded areas, essentially restoring them back to the control levels. Sc-236 showed the opposite effect on the expression of the late differentiation marker FLG (Figures 3D,E). The addition of the inhibitor suppressed the radiation-induced overexpression of FLG in both directly exposed and neighboring regions of the 3D cultures which was statistically significant for the 2 Gy exposed with and without inhibitor samples (Figure 3E).

### Radiation Effects in Organotypic Skin Cultures after NF-κB Inhibition

**Figure 4:**
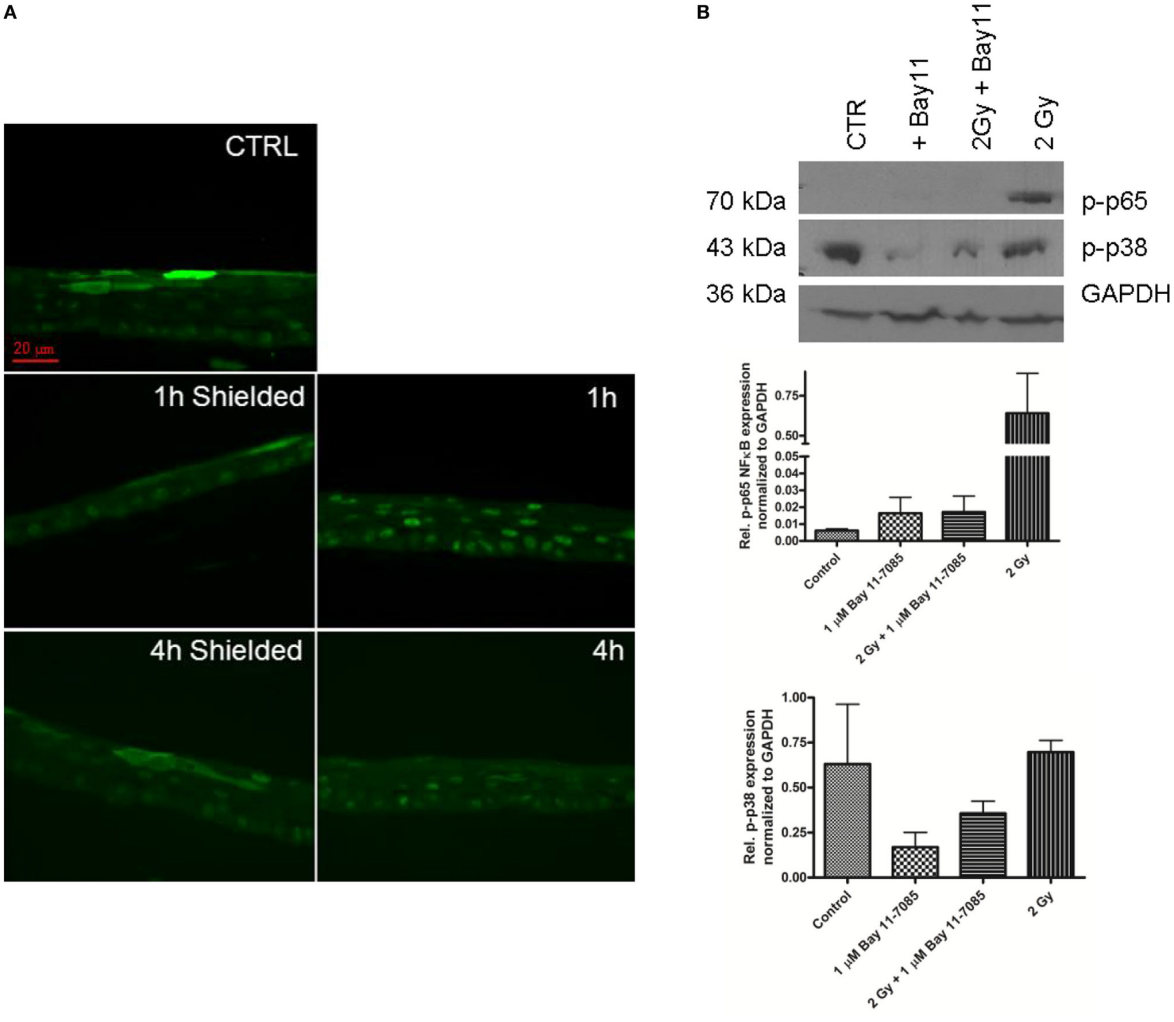
**NF-κB phospho-p65 formation 1–4 h after 2 Gy irradiation of 3D skin model**. Nuclear translocation 1–4 h postirradiation detected by immunofluorescence **(A)**. Green—p-p65 stain. Western blot analysis 1 h postirradiation shows high levels of p-p38 in the irradiated samples **(B)**. The addition of 1 µmol/l Bay 11-7085 is suppressing the p-p65 formation **(A,B)**. The graphs **(B)** represent the relative expression of p-p65 and p-p38 normalized to GAPDH detected by western blotting 1 h postirradiation. Results are arithmetical mean from two independent experiments. Each condition in these experiments had two replicate samples; error bars—SEM.

Nuclear factor kappa B is the major transcription factor that is responsible for the activation of COX-2 (16, 17). We measured the levels of the p65 NF-κB subunit that was phosphorylated on Ser276. First, we explored if irradiation of the 3D organotypic skin cultures induced phosphorylation and activation of p65 followed by its translocation into the nucleus. The immunofluorescence images showed translocation in the nucleus of p-p65 at 1 h postirradiation and still detectable 4 h after the broad field exposure of the cultures to 2 Gy 225 kVp X-rays (Figure 4A). However, this response was detected only in the irradiated parts of the 3D cultures, with the neighboring areas showing less abundant nuclear translocation. The early effect in the irradiated cells suggests that NF-κB phosphorylation and nuclear translocation precedes the activation of COX-2.

**Figure 5:**
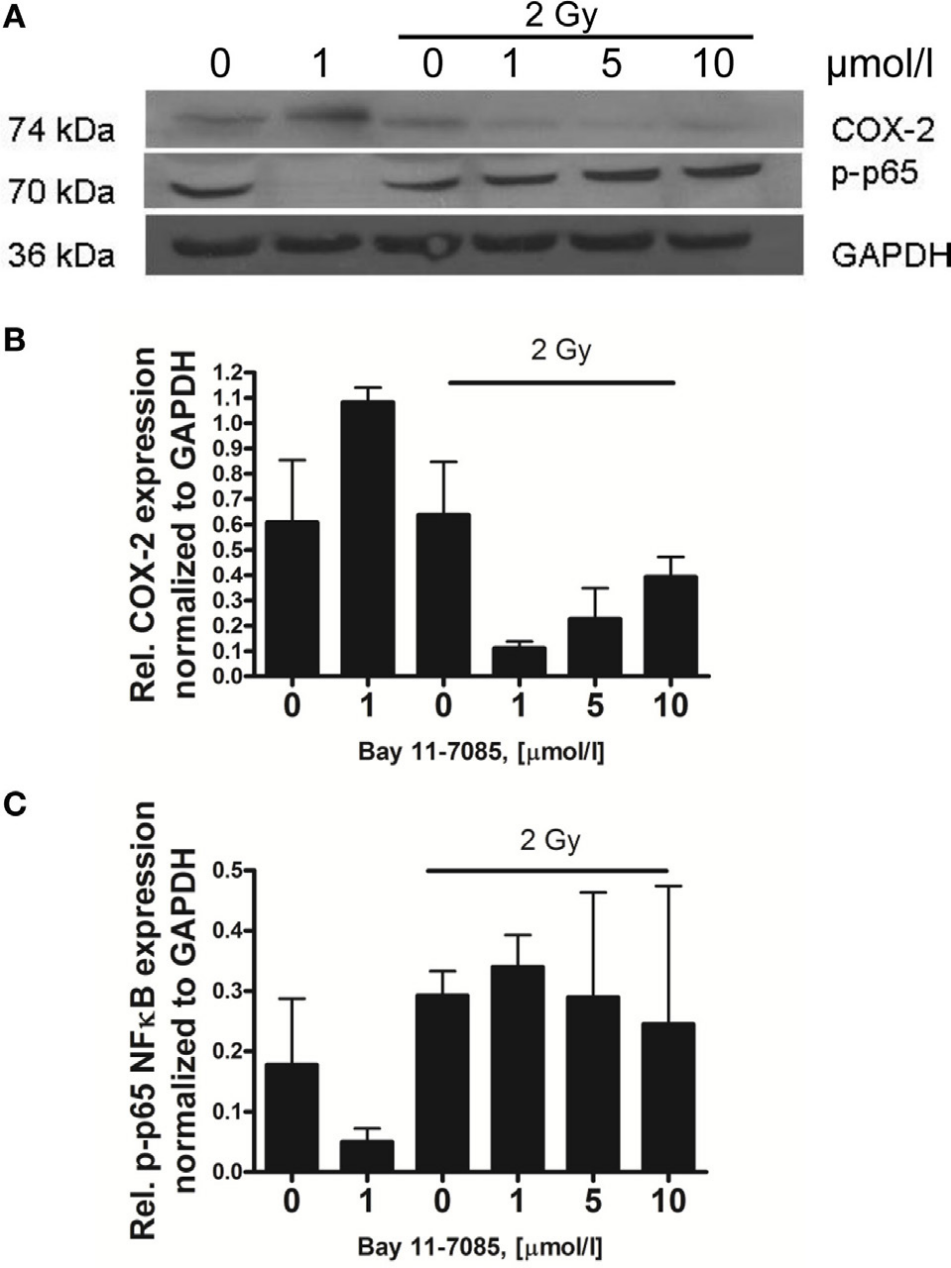
**Effect of Bay 11-7085 on cyclooxygenase-2 (COX-2) and p-p65 expression 4 h postirradiation of 3D organotypic skin cultures**. The 1 µmol/l treated samples show the effect of the inhibitor on the COX-2 and p-p65 protein levels **(A)**. The last three samples on each graph are increasing concentrations of the inhibitor plus 2 Gy 225 kVp X-ray irradiation. The positive irradiated control is 2 Gy. Graphs **(B,C)** represent the relative protein expression normalized to GAPDH. Results are arithmetical mean from two independent experiments. Each condition in these experiments had two replicate samples; error bars—SEM.

Furthermore, we confirmed, *via* western blotting, the radiation-induced phosphorylation of p65 in the skin samples 1 h post 2 Gy irradiation (Figure 4B). We also tested the specific NF-κB inhibitor Bay 11-7085 to prevent the radiation-induced activation of the transcription factor (Figure 4B). The optimal non-toxic concentration of Bay 11-7085 was first tested on N/TERT-1 cells (Figure 2B). The Bay 11-7085 had higher toxicity than sc-236 as the application of the inhibitor at 5 µmol/l concentration led to a statistically significant reduction of cell viability (Figure 2B). Based on these data, we tested 1 µmol/l Bay 11-7085 on the 3D cultures for inhibition of p65 phosphorylation (Figure 4B). It was proven that Bay 11-7085 suppresses the p65 phosphorylation and also reduces the phosphorylation of p38 (Figure 4B), suggesting that there is a connection between the p-p65 formation and the p-p38 levels. NF-κB is one of the main transcription factors responsible for COX-2 upregulation in skin (34). After the observation that the p65 subunit of NF-κB is being phosphorylated and translocated into the nucleus 1 h postirradiation, the next step in our experiments was to determine if the addition of NF-κB inhibitor blocks the COX-2 activation. Western blots were performed to detect if the NF-κB inhibition influences COX-2 expression (Figures 5A,B).

The addition of 1 µmol/l Bay 11-7085 to the culture medium of the 3D skin model induced complete disappearance of the p-p65 4 h postirradiation (Figures 5A,C). The combination of the NF-κB inhibitor and 2 Gy irradiation did not have an effect on the p-p65 levels, as these were upregulated 1 and 4 h postirradiation even at the highest concentration of the inhibitor used (Figures 4B and 5A,C). An important observation was the effect of 1 µmol/l Bay 11-7085 on the expression of COX-2. All the tested doses of NF-κB inhibitor downregulated the COX-2 expression after the 2 Gy treatment (Figure 5B).

### Morphology of the 3D Cultures after Bay 11-7085 Treatment

**Figure 6:**
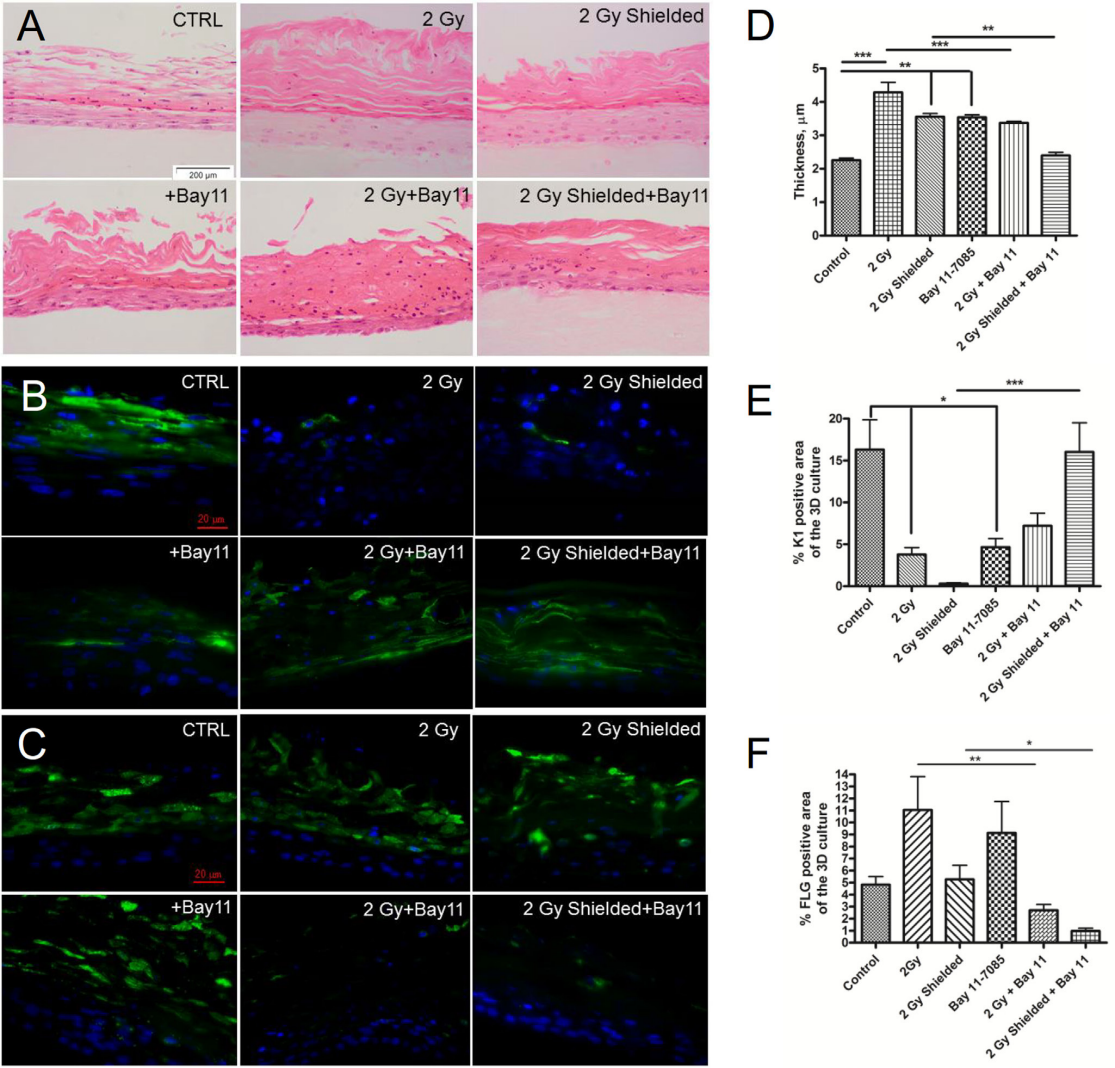
**H&E staining—morphological analysis of the 3D cultures after irradiation and incubation of the 3D epidermal model with 1 µmol/l specific NF-κB inhibitor for a period of 7 days (A)**. Cytokeratin 1 **(B)** and filaggrin (FLG) **(C)** expression in 1 µmol/l Bay 11-7085 treated 3D organotypic skin cultures 7 days after irradiation. Changes in the cornified layer thickness are calculated by Image J **(D)**. Quantification of K1 and FLG expression in the same 3D cultures by Image J as described in Section “Materials and Methods” **(E,F)**. Blue—DAPI; green—K1. Results are arithmetical mean from two independent experiments. *p < 0.05; **p < 0.01; ***p < 0.001, one-way ANOVA analysis, Tukey posttest.

In our studies, we also investigated morphological changes in the 3D cultures in the presence of 1 µmol/l Bay 11-7085 during the 7 days incubation period postirradiation. The inhibitor only treated cultures had both an increased density of the cornified layer and significant increase of the thickness of the cornified layer, but in general normal stratification (Figures 6A,D). The directly exposed regions had disrupted cornification, and there were numerous nuclei observed in the outer layers of the model (Figure 6A). In the half-shielded cultures irradiated with 2 Gy and treated with Bay 11-7085 H&E staining, there were also visible morphological changes. The NF-κB inhibition appeared to reduce the radiation-induced thickening of the cornified layer in both directly exposed and bystander areas of 3D skin cultures. Despite this positive effect, the overall morphology and thickness of Bay 11-7085 treated and irradiated 3D cultures showed large differences from the control (Figure 6A).

Additional to the H&E analysis, we also performed analysis and quantification of K1 and FLG expression in order to investigate the stages of the differentiation process in Bay 11-7085 treated samples. First, K1 expression was evaluated. The inhibitor by itself led to a statistically significant reduction in the levels of K1 (Figures 6B,E) as quantification by Image J confirmed. The combination of 2 Gy IR and 1 µmol/l Bay 11-7085 treatment showed a statistically significant rescue of the K1 levels in the shielded areas of the cultures. However, the effect was not significant in the directly irradiated parts. The increase in K1 levels in the inhibitor-treated samples was considerably lower than the control and not statistically different from the irradiated only samples (Figures 6B,E).

The effect of the Bay11-7085 on the morphology and expression of differentiation markers in the 3D cultures suggests that the inhibitor has a significant influence in changing the normal expression pattern of the early and late markers. In combination with IR, positive effects toward restoration of the normal tissue morphology were not observed and although there was partial rescue of the early differentiation marker K1, the complete reduction of FLG (Figures 6C,F) points to a substantial deviation from the normal differentiation pattern.

### Role of the Prostaglandin PGE2 in Skin Radiation Response

**Figure 7:**
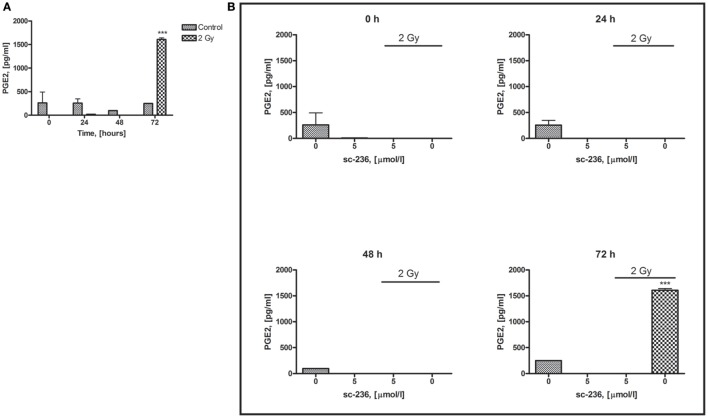
**Time dependence of PGE2 production in 3D organotypic skin cultures after 2 Gy irradiation (A) and effect of COX-2 inhibitor (5 µmol/l sc-236) on the secretion of PGE2 (B)**. The PGE-2 levels were measured at 0, 24, 48, and 72 h postirradiation. ***p < 0.001, one-way ANOVA analysis, Tukey posttest.

After observation of COX-2 upregulation in both directly irradiated and shielded areas of our 3D skin model and the inflammatory-like phenotype of the cultures, we further aimed to investigate if the PGE2 enzyme product of COX-2 is also upregulated and the timescale of this process. Initially, we performed a PGE2 Parameter™ assay (R&D Systems, Abingdon, UK) on 3D culture medium samples collected 2, 4, 6, and 24 h postirradiation (data not shown). We could not detect any changes in the PGE2. In later experiments, a longer timescale was investigated. Samples were analyzed at 0, 24, 48, and 72 h postirradiation (Figure 7A).

Constant low basal levels of PGE2 were detected in all control samples during the time course of the experiment. In the 2 Gy irradiated samples, where we observed the highest COX-2 gene and protein induction at 4 h postirradiation and the most-significant morphological changes of the skin cultures, PGE2 induction was not detected before 72 h. However, at 72 h postirradiation, the COX-2 product’s levels were significantly elevated (6.5 times higher than in the initial levels of the non-irradiated 3D cultures medium).

### COX-2 and NF-κB Inhibition to Control Prostaglandin Levels

One of our main aims was to find possible strategies for reduction of the radiation-induced signaling within the 3D skin model that is responsible for the early and late effects. In this part of the experimental work, we aimed to investigate if the mechanism of reduction of the radiation-induced epidermal effects is based on decrease of the main product of COX-2, PGE2.

We tested if the PGE2 levels in the tissue culture medium were decreased after treatment of the cultures with 5 µmol/l sc-236 specific COX-2 inhibitor 1 h prior to irradiation. A statistically significant increase in PGE2 levels was observed at 72 h postirradiation. The inhibitor reduced both the control levels of PGE2 in all samples and completely inhibited the prostaglandin synthesis by X-rays at 72 h (Figure 7B), suggesting that the prevention of the morphological changes in the 3D organotypic cultures postirradiation is mainly *via* suppression of PGE2 production.

## Discussion

### COX-2 Inhibitor and the Control of Radiation-Induced Reactions in the 3D Skin Model: Possible Applications of the COX-2 Inhibitor in Radiotherapy

In order to find possible ways to control the inflammatory-like skin responses induced by IR, we used the COX-2 selective inhibitor sc-236 at concentrations that considerably reduced the COX-2 expression (Figures 1 and 3). The effects of sc-236 were observed at transcriptional and translational level. After proving the inhibitory effect of sc-236, we aimed to reveal if its application would lead to suppression of the morphological changes that we observed in the irradiated and shielded 3D cultures. Experiments showed that COX-2 inhibition rescued the normal phenotype of the 3D cultures and hypercornification was reduced (Figure 3). More importantly, expression of differentiation markers returned to levels comparable with the control (Figures 3B–E). Similar effects were observed in the shielded areas of the 3D cultures, suggesting that the blocking of COX-2 functions also reduces the signals toward the non-irradiated areas and suppresses the bystander effects in the 3D skin model. The restoration of the expression levels of K1 and the late marker FLG suggests formation of functional epidermis with preserved barrier functions and without late consequences of the exposure to the IR (27, 35, 36). There was FLG upregulation from the inhibitor that could be explained with accelerated processing of the FLG precursor after COX-2 inhibition. COX-2 has previously been reported to suppress FLG expression (37) and its inhibition might result in increased levels of FLG. However, studies with FLG overexpressing mice have shown that they do not have any defects in keratin folding, and the skin barrier function recovers more effectively after external insult (38).

All observations of COX-2 inhibitor effects in the 3D skin model imply that it could be used in the clinic for reduction of the normal skin effects after radiotherapy. Anti-inflammatory drugs, such as corticosteroids, have already been utilized in breast cancer treatment with a positive outcome for patients manifested as reduction of radiation-induced skin effects (7). One of the important functions of the COX-2 inhibitors is that they specifically target cells with increased COX-2 levels (14). COX-2 is induced in inflammatory tissue reactions, but it has also been reported to be upregulated in various tumor cell lines such as glioma, adenocarcinoma, and breast cancer (13, 14). According to these data, inhibition of COX-2 could increase the radiosensitivity of cancer cells. Consequently, the enzyme could be a suitable target during radiotherapy for both an increase in radiosensitivity of the tumor cells and a decrease of normal tissue reactions (39). Despite the expected positive effects, the majority of the COX-2 inhibitors have unexpected severe side effects (11, 13) and each one should be carefully tested before considering its clinical use. The sc-236 that has been used in our study showed low normal tissue cytotoxicity, has already been applied in preclinical studies as an anti-cancer treatment (20), and could be a promising anti-inflammatory drug in the treatment of radiation-induced skin inflammatory reactions.

### NF-κB Inhibitor and Effect on the Radiation-Induced Responses in the 3D Model

In a further attempt to reduce the skin model response to IR, we used an inhibitor of the transcription factor NF-κB (Figures 4–6). The role of NF-κB as a key element for radiation-induced inflammation and its antiapoptotic function have been previously highlighted as potential targets for enhancement of the cell kill during radiotherapy treatments (16, 19, 22, 40). The p-Ser-276 modification of NF-κB is one of the major active forms of the transcription factor, induced from inflammatory stimuli such as TNF-α and LPS (16). NF-κB is activated by inflammatory cytokines and the process of signal transmission between the cytokine receptors and the intracellular transcription factor is regulated *via* phosphorylation of p-p38 MAPK (17). On the basis of these data and studies showing direct activation of NF-κB by IR (19), we investigated the involvement of this transcription factor in the pro-inflammatory responses observed in the 3D skin model. To test its role, we decided to use a specific inhibitor NF-κB (Bay 11-7085) that prevents the phosphorylation of the p65 unit of the transcription factor. The inhibitor was not able to suppress completely the radiation-induced p-p65 formation (Figure 4). The inhibitor has been found to downregulate COX-2 expression postirradiation (Figure 5). This was further evidence of the NF-κB → COX-2 link for the series of reactions activated by radiation in skin. It has been suggested that the p38 MAPK is an intermediate player between these two molecules (40, 41) and in our studies the use of the NF-κB inhibitor reduced p-p38 phosphorylation (Figure 4B). It suggests also that Bay 11-7085 is not totally specific for suppressing p65 phosphorylation but also affects other targets. The phosphorylation of p38 might be switching on a cascade of phosphorylation reactions leading to long-term activation of NF-κB. However, if this is the connecting link or there is an intermediate transducer, it needs to be further confirmed.

Regarding the morphological effect, the use of the NF-κB inhibitor showed no evidence of being able to rescue the normal phenotype (Figure 6). On the contrary, the inhibitor had a detrimental effect on the 3D cultures similar to that induced by radiation. A possible explanation of these observations is that since NF-κB is an antiapoptotic factor, its inhibition causes an increase of both apoptotic death and development of harmful effects in normal tissue (22). The effects of the inhibitor on the expression of differentiation markers were also unfavorable. Bay 11-7085 decreased K1 levels and up-regulated FLG (Figure 6). Similar effects were observed after radiation treatment only. The combination of radiation and NF-κB inhibition led to partial restoration of K1 in the directly irradiated areas and complete rescue of K1 levels in the shielded parts of the 3D cultures. Although there was a positive effect from the inhibitor on the expression of the early differentiation marker, the late marker FLG was drastically downregulated. This suggests abnormal differentiation, which possibly affects the normal functional properties of the 3D skin cultures (35). Since the inhibition of COX-2 had a restoring effect on the 3D culture morphology and differentiation and the NF-κB inhibition reduces COX-2 expression, it could be expected that NF-κB inhibition also would have a positive effect on the *in vitro* skin model postirradiation. Interestingly, we did not observe such rescue, but the opposite—negative effects on the 3D culture development in the presence of inhibitor. This could be explained by the crucial role of NF-κB in important epidermal processes such as cellular growth and homeostasis, epidermal proliferation, and differentiation (34). NF-κB p65 nuclear translocation has been highlighted as an important factor in the exit of the basal cells from the cell cycle that pushes them toward terminal differentiation (42). The same authors suggested that NF-κB inhibition could induce epidermal hyperplasia by blocking this mechanism of basal cell cycle exit. This could be the reason for the increased thickness of the cornified layer in Bay 11-7085 treated cultures. Even though NF-κB inhibition is thought to have an anti-inflammatory effect, in combination with its hyperproliferation inducing capacity in skin, the overall effect for the tissue response tends to be negative.

### Role of PGE2 Prostaglandin in Radiation-Induced Responses in 3D Skin Model

The inflammatory-like cascade triggered by IR in 3D skin includes long distance-acting signaling molecules. We focused our attention on the PGE2 prostaglandin produced by COX-2 since it has important role in the perpetuation and maintenance of the local inflammation (10, 34).

PGE2 upregulation was observed considerably later than the irradiation of the 3D cultures (Figure 7). The prostaglandin production was detectable and statistically significant over control levels at 72 h after exposure. This suggests that COX-2 and its product PGE2 are more likely to make a strong contribution to the persistence of the pro-inflammatory changes of the 3D epidermal model, rather than at the early stage of signal transduction. Moreover, COX-2 has previously been described to play an important role in the completion of the differentiation process and mice with overexpression of COX-2 have been shown to develop abnormally differentiated epidermis (43). Here, we note that the basal levels of PGE2 postirradiation or after the inhibitor treatments has been very low, below control levels. The reason for this is not clear and further work is required. Furthermore, we investigated if the inhibition of COX-2 has a substantial effect on the release of PGE2 (Figure 7). When using the sc-236 inhibitor, a total reduction of radiation-induced PGE2 production at 72 h postirradiation was observed. This observation and the rescue of the normal phenotype and differentiation pattern of the 3D skin model support the hypothesis that the COX-2 enzyme *via* its product PGE2 is responsible for the late radiation-induced reactions in the 3D organotypic skin cultures.

The model used in these experiments has many advantages including its ability to differentiate and produce cytokines and prostaglandins upon pro-inflammatory stimuli. Although an interspecies model, it has reliable and reproducible differentiation abilities that have been used in our experiments. However, it should be kept in mind that this system also has limitations, especially when considering immune responses. Further experiments with more complicated models, involving an immune cell compartment are needed to extend the knowledge in these areas.

In conclusion, the use of inhibitors for reduction of side effects of radiation and the potential application in radiotherapy should be approached with caution. From the two anti-inflammatory inhibitors that have been tested, the COX-2 inhibitor seems a good candidate for the reduction of normal tissue effects, without affecting tissue differentiation and morphology. The NF-κB inhibitor on the other hand, despite reducing some of the radiation-induced morphological features (especially in the bystander areas), had a detrimental effect on differentiation markers expression and possibly on the 3D skin culture barrier function.

## Author Contributions

AA designed and performed the experiments and wrote the manuscript. GS and KP helped with the experiment design and contributed to the manuscript writing.

## Conflict of Interest Statement

The authors declare that the research was conducted in the absence of any commercial or financial relationships that could be construed as a potential conflict of interest.
