# Supplementary figures and images for: Pro-inflammatory Signaling in a 3D Organotypic Skin Model after Low LET Irradiation—NF-κB, COX-2 Activation, and Impact on Cell Differentiation
Source: Front Immunol. 2017 Feb 10;8:82. doi: 10.3389/fimmu.2017.00082 (PMC5300980; doi:10.3389/fimmu.2017.00082)

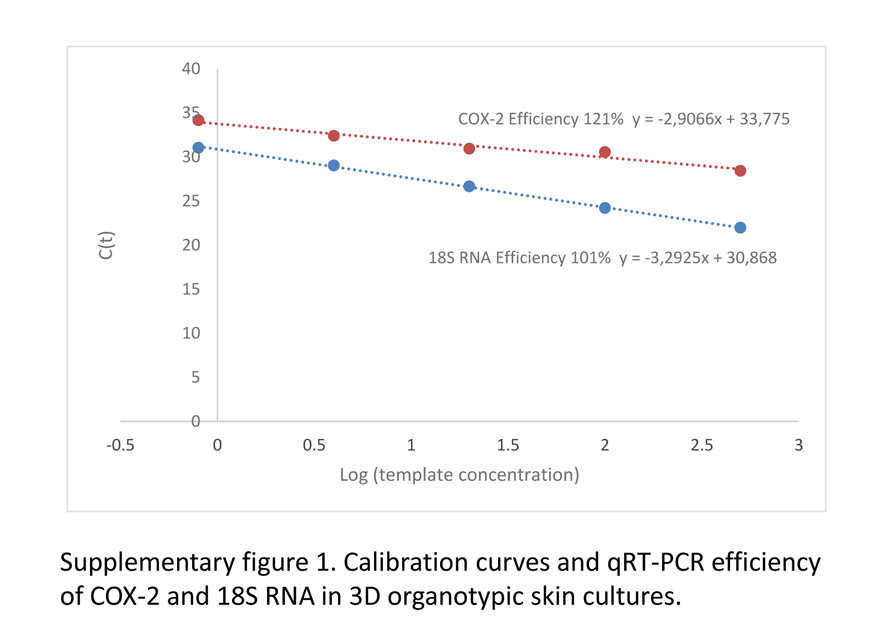

Supplement: Supplementary file 1 [file image_1.tif]
